# Supplementary material for: Novel loading protocol combines highly efficient encapsulation of exogenous therapeutic toxin with preservation of extracellular vesicles properties, uptake and cargo activity
Source: Discov Nano. 2024 Apr 30;19(1):76. doi: 10.1186/s11671-024-04022-8 (PMC11063024; doi:10.1186/s11671-024-04022-8)

Supplementary Material

Novel loading protocol combines highly efficient encapsulation of exogenous therapeutic toxin with preservation of extracellular vesicles properties, uptake and cargo activity

Stefania Zuppone[1], Natasa Zarovni[2], Kosuke Noguchi[3], Francesca Loria[2], Carlo Morasso[4], Andres Lohmus[2], Ikuhiko Nakase[3], Riccardo Vago[1,5]

[1] *Urological Research Institute, Division of Experimental Oncology, IRCCS San Raffaele Scientific Institute, Milano, 20132, Italy.*
[2] *HansaBiomed Life Sciences, Tallinn, 12618, Estonia*
[3] *Department of Biological Science, Graduate School of Science, Osaka Prefecture University, Osaka, 599-8531, Japan*
[4] *Istituti Clinici Scientifici Maugeri IRCCS, Pavia, 27100, Italy*
[5] *Università Vita-Salute San Raffaele, Milano, 20132, Italy*

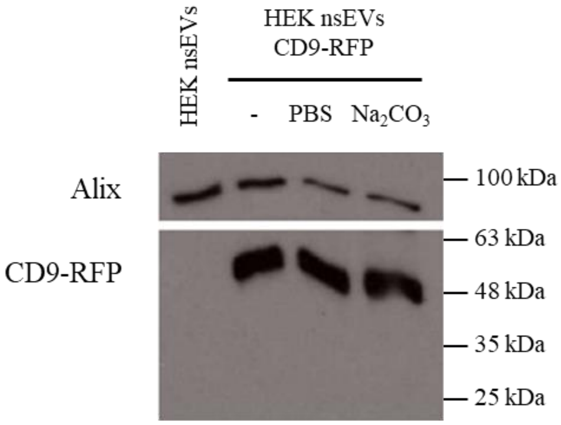

Fig. S1. HEK293 derived nsEVs structural stability upon sodium carbonate treatment. Western blot analysis Alix and RFP from untreated (-), PBS treated and sodium carbonate (Na2CO3) treated nsEVs derived from CD9-RFP expressing HEK293 cells. HEK293 WT derived nsEVs were used as negative control for CD9-RFP expression. CD9-RFP has been detected using anti-RFP antibody.

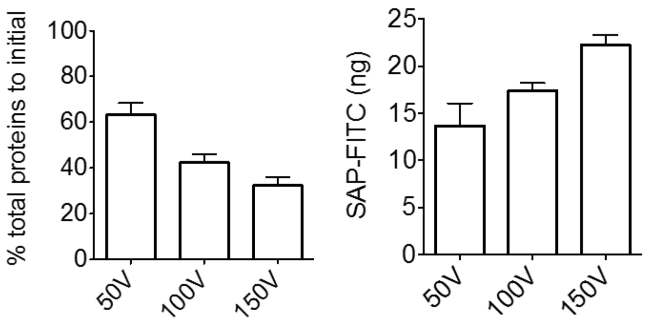

Fig. S2. Protein recovery and encapsulation efficiency evaluation after increasing voltages exposition and higher nsEVs:SAP-FITC ratios. A. BCA assay for quantification of total protein recovery after co-incubation of HEK293-derived nsEVs (25 ug/ml) with SAP-FITC (1:2 molecular ratio) and exposition to 50, 100, 150 V electroporation (see material and methods). Results are shown as percentage to initial. B. Quantification of SAP-FITC incorporation after incubation with HEK293-derived nsEVs (25 ug/ml) at a 1:2 molecular ratio and 50, 100, 150 V electroporation (see Material and Methods) by spectrofluorometer analysis.

Full blots

Fig. 1C

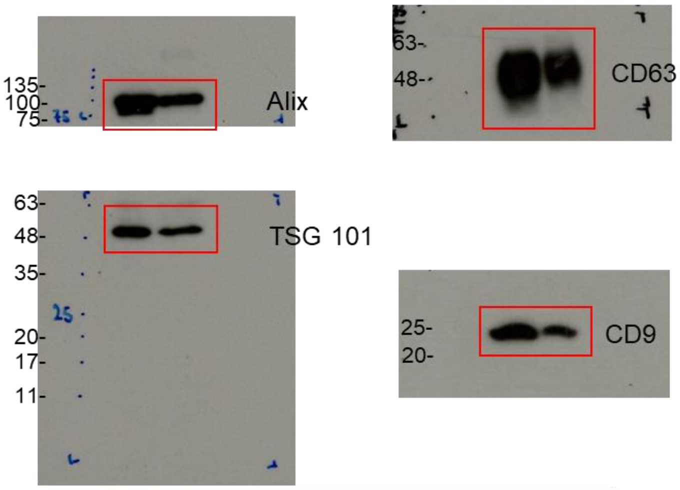

Fig. 6A

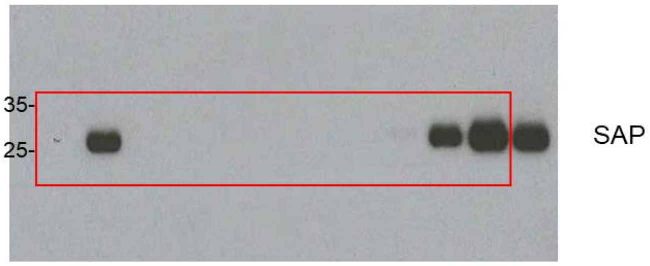

Sup. Fig. 1

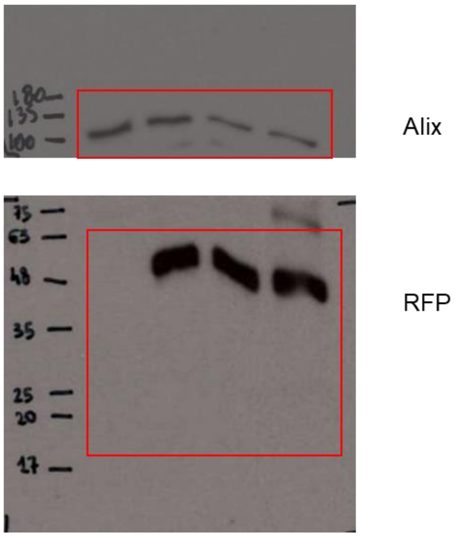

Supplement: Supplementary file 1 — Additional file 1. [file 11671_2024_4022_MOESM1_ESM.pdf]
